# Supplementary figures and images for: A chromosome-scale genome assembly of mungbean (Vigna radiata)
Source: PeerJ. 2024 Dec 23;12:e18771. doi: 10.7717/peerj.18771 (PMC11670757; doi:10.7717/peerj.18771)

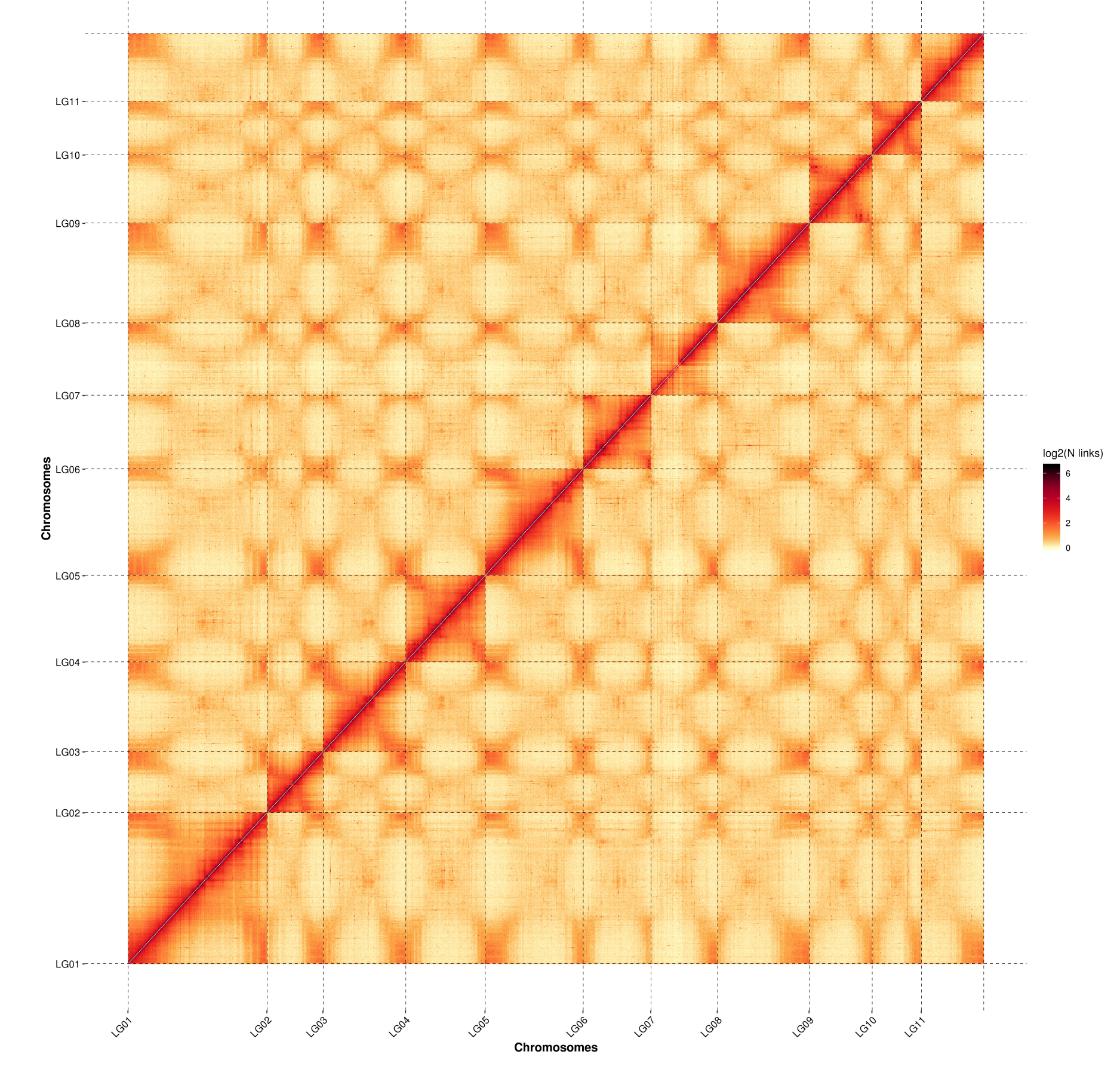

Supplement: Supplemental Information 1 [file peerj-12-18771-s001.png]

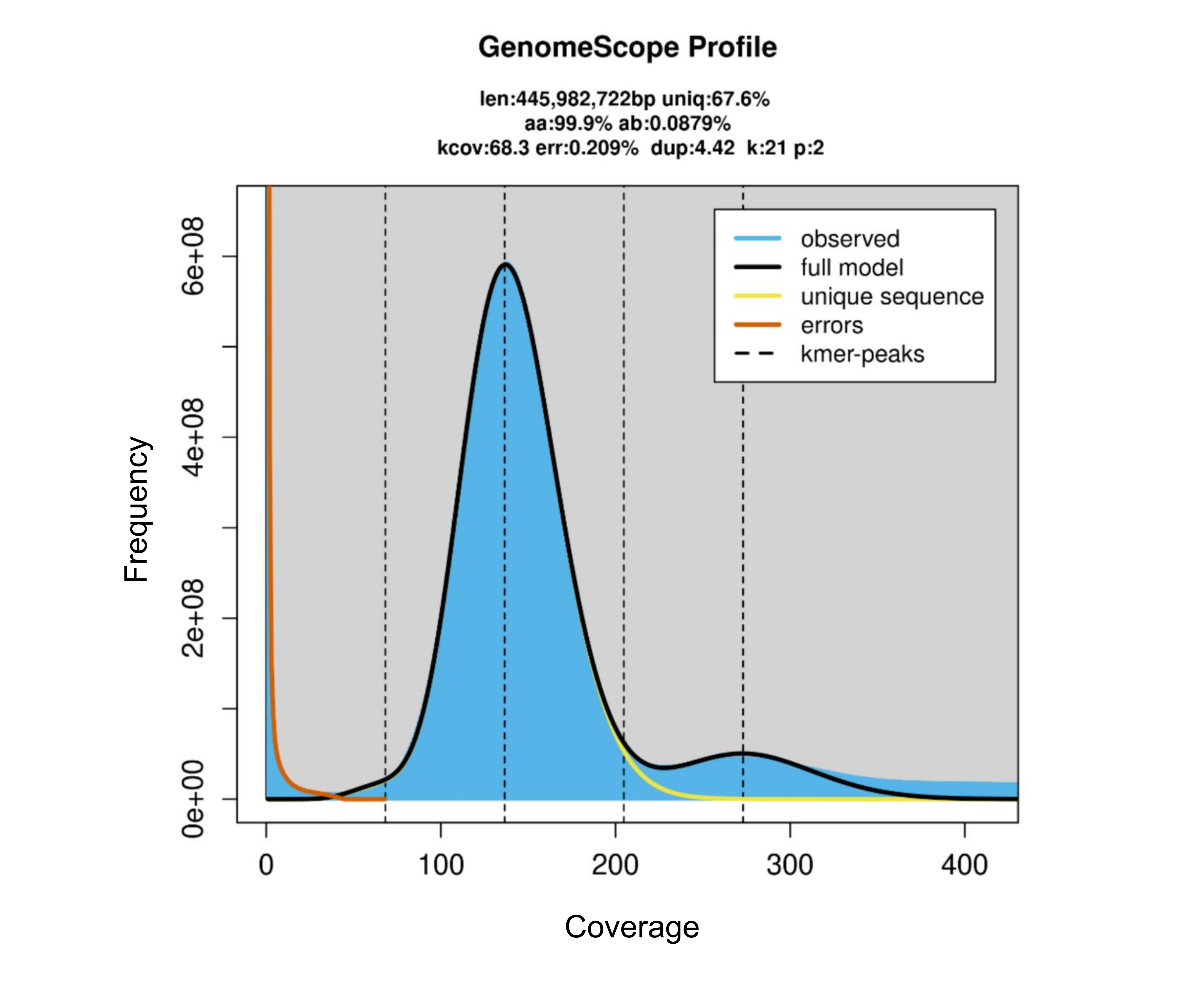

Supplement: Supplemental Information 2 [file peerj-12-18771-s002.png]

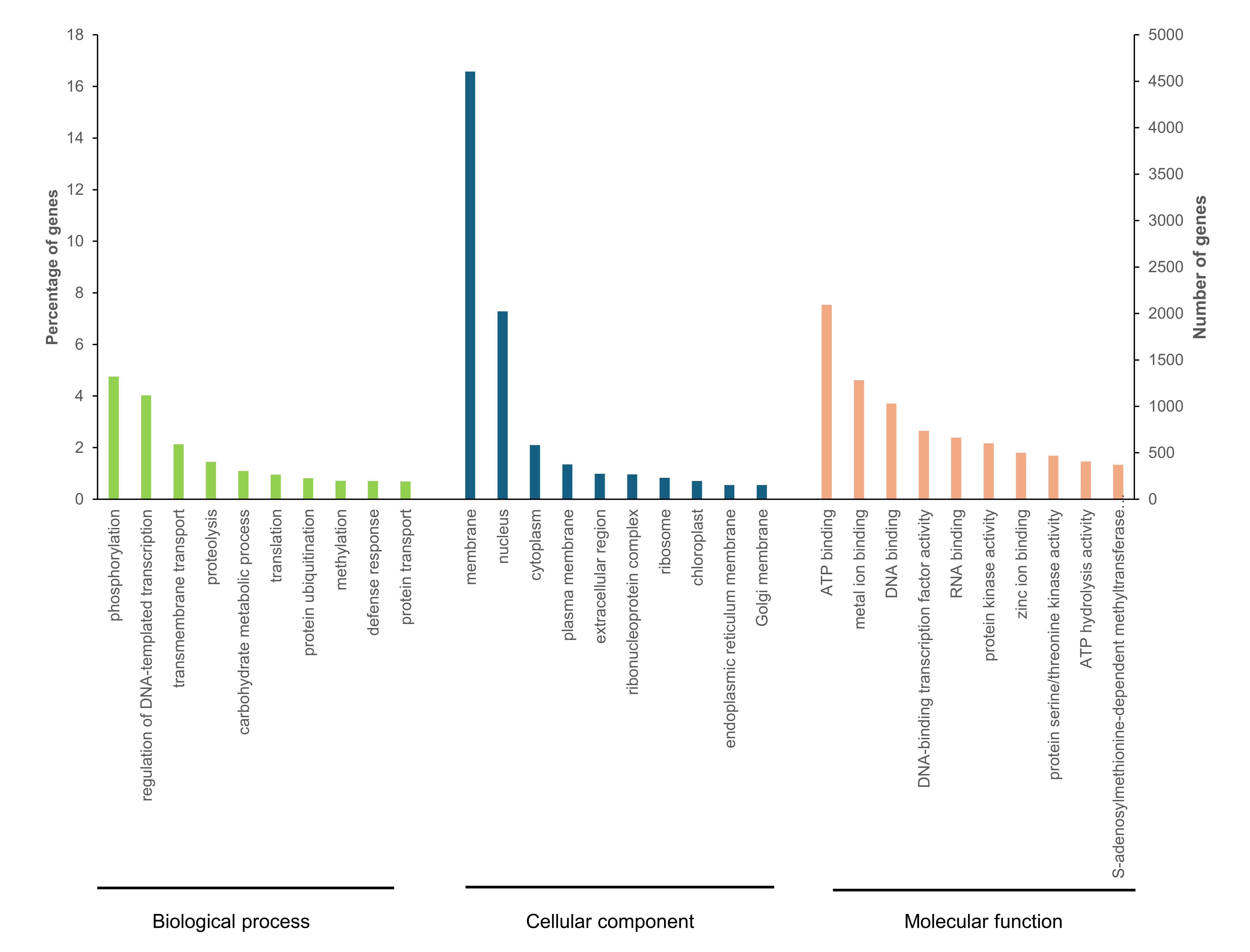

Supplement: Supplemental Information 3 [file peerj-12-18771-s003.png]
